# Hybrid Telemedicine–House-Call Model for Pneumonia Management in North Texas: Retrospective Pilot Study

**DOI:** 10.2196/98767

**Published:** 2026-09-16

**Authors:** Raika Bourmand, Trevor Lee, Prabha Nair, Saji Pillai

**Affiliations:** 1 Burnett School of Medicine at Texas Christian University Fort Worth, TX United States; 2 Department of Biology The University of Texas at Arlington Arlington, TX United States; 3 Paris Regional Health Paris, TX United States

**Keywords:** telemedicine, hybrid care model, pneumonia, emergency department use, digital health, care delivery innovation, health services research, pilot study

## Abstract

**Background:**

Pneumonia accounts for approximately 1.4 million emergency department (ED) visits annually in the United States. Hybrid care models integrating telemedicine with in-home clinical support may offer a novel approach to managing appropriately selected patients while optimizing ED use.

**Objective:**

This study aimed to evaluate a hybrid house-call model for managing adults with pneumonia by describing patient characteristics, use patterns, illness severity, and ED referral rates.

**Methods:**

We conducted a retrospective descriptive case-series study among adults diagnosed with pneumonia within a primary and urgent care system between January 1, 2022, and January 1, 2024. Cases were identified using the *International Classification of Diseases, 10th Revision* (*ICD-10*) codes (J18.9, J15.8, J16.8, J17, and J18.1). In total, 30 patient records were screened, and 28 met the eligibility criteria for analysis. The hybrid model combined virtual physician evaluation with in-home assessment by a trained health care professional using digital diagnostic tools. Data collected included visit type, treatments, comorbidities, CRB-65 (confusion, respiratory rate, blood pressure, age ≥65 years) scores, and ED referrals. Differences between patients referred to the ED and those who were not referred to the ED were evaluated using the Mann-Whitney *U* test.

**Results:**

In total, 28 patients accounted for 56 clinical encounters (median 2, IQR 1-3 encounters per patient). CRB-65 scores ranged from 0 to 2, with 13 (46.4%) patients scoring 0, 13 (46.4%) patients scoring 1, and 2 (7.1%) patients scoring 2. Of the 28 patients, 8 (28.6%) were referred to the ED. Patients referred to the ED had significantly higher CRB-65 scores than those managed without ED referral (median 1, IQR 1-1.5 vs median 0, IQR 0-1; Mann-Whitney *U*=34.0; *P*=.01).

**Conclusions:**

This retrospective descriptive case-series study demonstrates the implementation of a hybrid telemedicine–house-call model for adults with pneumonia in a real-world clinical setting. Larger prospective comparative studies with longitudinal follow-up are needed to evaluate clinical outcomes, referral performance, safety, and effectiveness.

## Introduction

Telemedicine, defined as the remote delivery of health care via telecommunications technology, has evolved since the 20th century but experienced exponential growth during the COVID-19 pandemic, increasing by nearly 766% in the first 3 months [1]. During this time, public health measures and fear of infection led many patients to avoid traditional health care settings, accelerating the adoption of virtual care [2]. As digital infrastructure has improved, telemedicine has emerged as a key tool for increasing health care access, particularly for patients with disabilities, those in rural or underserved communities, and individuals facing barriers to transportation [3,4]. As a result, telemedicine has become a core component of digital health delivery, enabling alternative models of care that extend beyond traditional clinic settings.

Beyond improving access, telemedicine offers increased convenience and cost-efficiency. It enables patients, especially older adults and those with chronic conditions, to engage with health care providers from their homes, reducing the need for in-person visits for follow-up care or minor health concerns [5]. This approach facilitates chronic disease management through remote monitoring tools and has been shown to reduce preventable emergency department (ED) visits, particularly in vulnerable populations [6,7]. For example, a study conducted by the Collaborative for Telehealth and Aging to identify telehealth guidelines for older adults reported a significant association between poor chronic disease management and ED use among older adults, suggesting that telemedicine may help mitigate this issue [8]. Furthermore, telemedicine has played a crucial role during crises such as the COVID-19 pandemic and natural disasters, maintaining continuity of care while minimizing transmission risks [9].

Despite these benefits, telemedicine poses challenges, including limited ability to collect vital signs and perform physical examinations. To address these limitations, hybrid telemedicine–house-call models have emerged as an early-stage care delivery innovation [10]. This approach combines virtual consultation with in-person assessment by a medical assistant or nurse at the patient’s home, enabling real-time recording of vital signs, point-of-care testing, and use of digital diagnostic tools such as stethoscopes and otoscopes [10]. The hybrid model not only expands the clinical capabilities of telemedicine but also provides insight into the patient’s home environment, facilitating rapport and more holistic care [11]. However, despite the growing implementation of hybrid house calls, little research has evaluated their clinical impact, particularly in reducing avoidable ED visits for common but potentially serious conditions such as pneumonia.

Community-acquired pneumonia is an acute respiratory infection characterized by inflammation of the alveoli in one or both lungs, often resulting in accumulation of fluid or purulent material. Etiologic agents include bacteria, viruses, and fungi, with clinical manifestations such as productive or nonproductive cough, fever, chills, and dyspnea. The severity of illness varies depending on host factors, pathogen virulence, and comorbid conditions [12]. In 2021, approximately 1.2 million ED visits in the United States were attributed to pneumonia caused by an infectious organism as the primary diagnosis [13].

This study presents a retrospective pilot evaluation of a hybrid telemedicine–house-call model for the management of community-acquired pneumonia. The study aimed to evaluate this model through a descriptive case-series study of adults with pneumonia by describing patient characteristics, use patterns, illness severity, and ED referral rates in a real-world clinical setting.

## Methods

### Ethical Considerations

This study was reviewed and approved by the Texas Christian University Institutional Review Board (IRB; IRB #2023-422). The requirement for informed consent was waived because this was a retrospective chart review involving existing electronic health record (EHR) data with no direct patient contact and minimal risk to participants. All study procedures were conducted in accordance with the ethical principles of the Declaration of Helsinki and applicable institutional and regulatory guidelines.

### Study Design and Setting

We conducted a retrospective descriptive case-series study evaluating a hybrid house-call model for the management of adults with pneumonia. EHRs from a tertiary care center and affiliated hybrid house-call practice in North Texas were reviewed for encounters occurring between January 1, 2022, and January 1, 2024. The primary objective was to assess outpatient pneumonia management through this care model by describing patient characteristics, use patterns, and ED referral rates. Patient records were identified using the following *International Classification of Diseases, 10th Revision* (*ICD-10*) diagnosis codes related to pneumonia: J18.9 (pneumonia and unspecified organism), J15.8 (other bacterial pneumonia), J16.8 (pneumonia due to other specified infectious organisms), J17 (pneumonia in diseases classified elsewhere), and J18.1 (lobar pneumonia and unspecified organism). Eligible participants were adults (aged ≥18 years) with a documented diagnosis of pneumonia identified by one of the predefined *ICD-10* codes, who were evaluated through the hybrid house-call program during the study period. Patients aged <18 years or without sufficient documentation to confirm a pneumonia diagnosis were excluded. Records lacking sufficient information to confirm a pneumonia diagnosis were excluded prior to analysis. For the remaining cohort, analyses were conducted using complete-case data, and no imputation of missing values was performed. All data were handled in a secure manner, and only deidentified data were used for analysis to protect participant confidentiality.

### Hybrid House-Call Intervention

Patients received evaluation through a hybrid house-call model consisting of an in-home visit performed by a trained health care professional in conjunction with real-time physician oversight using telemedicine. Clinical assessment included medical history taking, physical examination, vital signs recording, and point-of-care evaluation when indicated. On the basis of clinical judgment, patients were managed at home with treatment, scheduled for follow-up, or referred to the ED when a higher level of care was deemed necessary.

### Participant Selection

All patient records were anonymized prior to analysis. Extracted variables included patient demographics (age and sex), the total number of visits per patient, visit type (hybrid house call vs other), treatments administered, comorbidities, CRB-65 (confusion, respiratory rate, blood pressure, age ≥65 years) score [14,15], ED referral, and rural residence. The primary outcomes were the proportion of patients successfully managed without ED referral and the use of the hybrid house-call model (total number of visits). Secondary outcomes included CRB-65 severity distribution, comorbidity burden, and the characteristics of patients requiring ED referral.

### Data Collection and Outcome Measures

Because blood urea nitrogen measurements were not consistently available at the index encounter, illness severity was assessed using the CRB-65 score rather than the CURB-65 (confusion, urea, respiratory rate, blood pressure, age ≥65 years) score. CRB-65 assigns 1 point each for new-onset confusion, respiratory rate ≥30 breaths per minute, systolic blood pressure <90 mm Hg or diastolic blood pressure ≤60 mm Hg, and age ≥65 years. Scores range from 0 to 4, with higher scores indicating greater predicted mortality risk. Clinical variables were abstracted from documentation recorded during the index clinical encounter, defined as the first eligible encounter during the study period in which one of the predefined pneumonia *ICD-10* diagnosis codes was assigned [16].

Data were entered into Microsoft Excel [17] for analysis. Descriptive statistics were used to summarize patient characteristics, the number of visits per patient, CRB-65 scores, ED referral rates, and treatment patterns. Continuous variables are reported as means (SDs) or medians (IQRs), as appropriate, and categorical variables are reported as frequencies and percentages. Frequency distributions were used to categorize CRB-65 scores. Rural residences were determined using the Health Resources and Services Administration Rural Health Grants Eligibility Analyzer [18]. Patients referred to the ED were further stratified according to comorbidities and CRB-65 scores to descriptively characterize referral patterns. The patient served as the primary unit of analysis. For patients with multiple encounters, visit-level data were aggregated to summarize health care use, while patient-level outcomes were analyzed once per individual.

### Statistical Analysis

Continuous variables are presented as means (SDs) or medians (IQRs), as appropriate. Categorical variables are presented as frequencies and percentages. Because CRB-65 scores were ordinal and not normally distributed, differences between patients referred to the ED and those managed without ED referral were evaluated using the Mann-Whitney *U* test. Statistical significance was defined as *P*<.05. Analyses were performed using Python (version 3.13.7) with the *SciPy* library.

## Results

### Participant Flow and Sample Demographics

A total of 30 patient records were screened for eligibility (Figure 1). Two records were excluded: one involved a patient aged <18 years and the other lacked sufficient documentation to confirm a pneumonia diagnosis. The final analytic cohort therefore included 28 adult patients. Of these, 8 (28.6%) were male and 20 (71.4%) were female. The mean age was 58.9 (SD 21.8) years. Of the 28 patients, 14 (50%) patients resided in areas classified as rural (Table 1).

**Figure 1:**
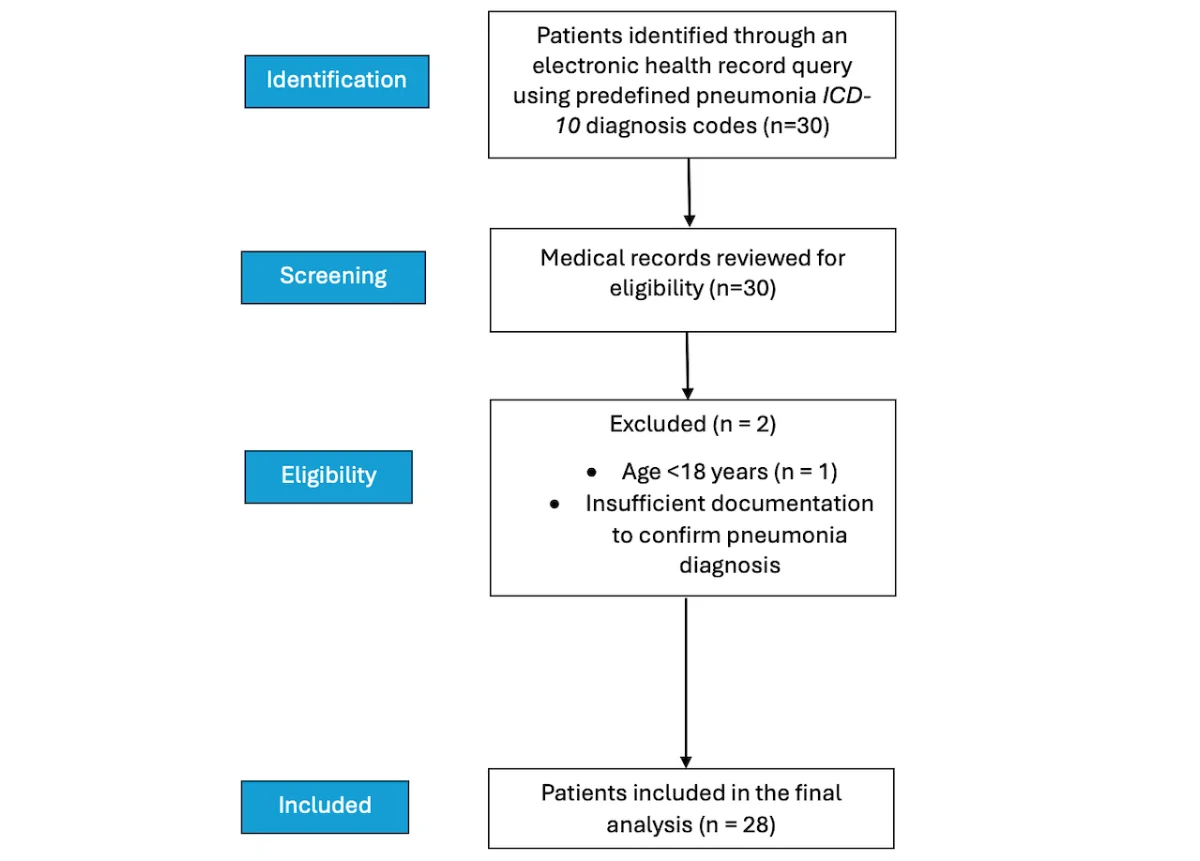
Study selection flow diagram of adults with pneumonia managed through a hybrid telemedicine–house-call model in North Texas. ICD-10: International Classification of Diseases, 10th Revision.

**Table 1:** Baseline characteristics of adults with pneumonia managed through a hybrid telemedicine–house-call model in North Texas (N=28)^a^.

Characteristics			Value
Age (years), mean (SD)			58.9 (21.8)
Sex, n (%)			
	Male	8 (28.6)	
	Female	20 (71.4)	
Rural residence, n (%)			14 (50)
Patients with ≥1 documented comorbidity, n (%)			21 (75)
Clinical encounters per patient, median (IQR)			2 (1-3)
Initial hybrid house-call visit, n (%)			24 (85.7)
Initial virtual visit, n (%)			3 (10.7)
Initial in-clinic visit, n (%)			1 (3.6)
Patients with ED^b^ referral, n (%)			8 (28.6)
Patients managed without ED referral, n (%)			20 (71.4)

^a^Total clinical encounters, n=56.

^b^ED: emergency department.

During the 2-year study period, 28 patients accounted for 56 clinical encounters. The median number of encounters per patient was 2 (IQR 1-3; range 1-7). Most patients initiated care through the hybrid house-call model (24/28, 85.7%), whereas 3 (10.7%) patients initiated care through a virtual visit and 1 (3.6%) patient initiated care through an in-clinic visit.

Of the 28 patients, 19 (67.9%) had documented insurance coverage, whereas 9 (32.1%) had no insurance on file. Of the 8 patients recommended for ED transfer, 2 were uninsured. Among the insured patients, 8 were covered through government-funded programs, including Medicare or WellMed [19], whereas 11 had private insurance coverage.

### CRB-65–Derived Mortality Risk and ED Referral

Over the 2-year study period, 28 patients accounted for 56 clinical encounters (median 2, IQR 1-3 encounters per patient). Of the 28 patients, 24 (85.7%) patients initiated care through the hybrid house-call model, 3 (10.7%) through virtual visits, and 1 (3.6%) through an in-clinic visit. In total, 8 (28.6%) patients were referred to the ED, all of whom had documented comorbidities.

CRB-65 scores ranged from 0 to 2. Overall, 13 (46.4%) patients had a CRB-65 score of 0, a total of 13 (46.4%) had a score of 1, and 2 (7.1%) had a score of 2. Patients referred to the ED had significantly higher CRB-65 scores than those managed without ED referral (median 1, IQR 1-1.5 vs median 0, IQR 0-1; Mann-Whitney *U*=34.0; *P*=.01; Figure 2 and Table 2).

**Figure 2:**
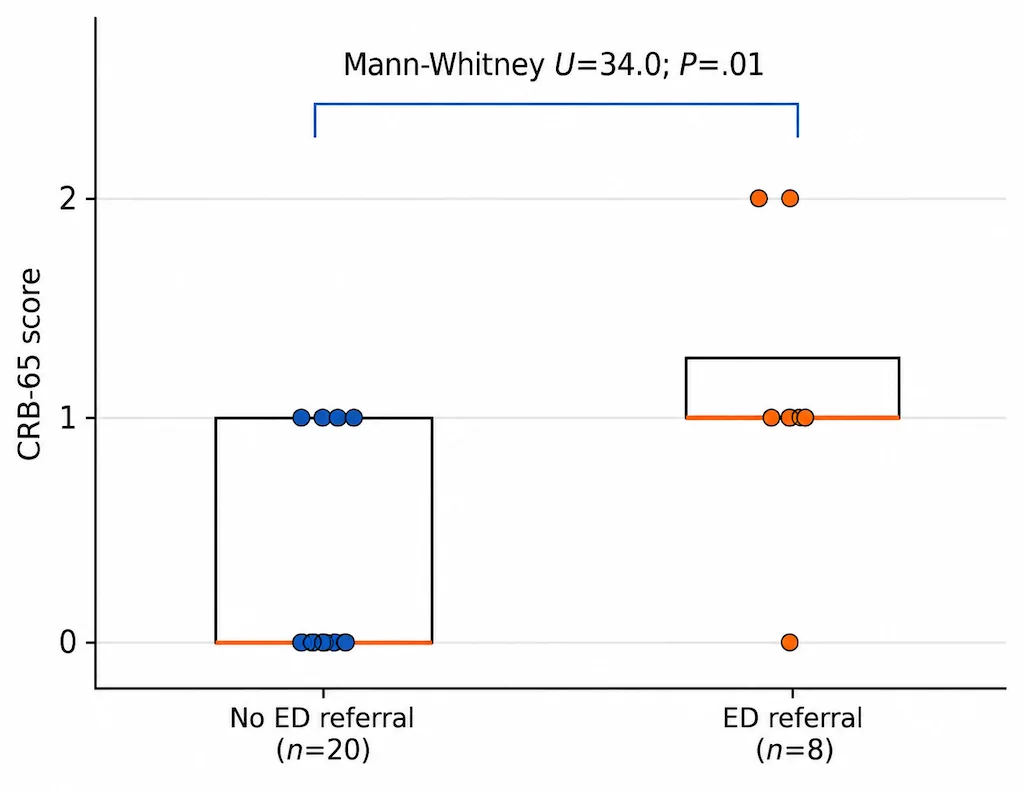
Distribution of CRB-65 (confusion, respiratory rate, blood pressure, age ≥65 years) scores by emergency department (ED) referral status among adults with pneumonia managed through a hybrid telemedicine–house-call model in North Texas. Box plots demonstrate higher CRB-65 scores among patients referred to the ED than among those managed without ED referral. The median CRB-65 score was 1 (IQR 1-1.5) in the ED referral group and 0 (IQR 0-1) in the non-ED referral group. Individual points represent observed patient values.

**Table 2:** Comparison of adult patients with pneumonia managed without emergency department (ED) referral versus those referred to the ED.

Characteristics	No ED referral (n=20)	ED referral (n=8)	*P* value
CRB-65^a^ score, median (IQR)	0 (0-1)	1 (1-1.5)	.01
Patients with ≥1 documented comorbidity, n (%)	13 (65)	8 (100)	.08

^a^CRB-65: confusion, respiratory rate, blood pressure, age ≥65 years.

## Discussion

### Principal Findings

In this retrospective descriptive case-series study, patients referred to the ED had significantly higher CRB-65 scores than those managed without ED referral, suggesting greater illness severity among patients requiring escalation of care. Most patients were managed without ED referral, whereas patients requiring escalation had higher illness severity and documented comorbidities. Although this pilot study was not designed to evaluate clinical outcomes or safety, these findings provide insight into ED referral patterns according to documented illness severity within the hybrid care model. These findings should be interpreted within the context of a small, real-world descriptive case-series study.

Our findings are consistent with previous literature demonstrating that alternative acute care models, including urgent care centers and telemedicine, may reduce ED use for lower-acuity conditions [20-23]. The hybrid house-call model extends this concept by combining in-home clinical assessment with physician oversight through telemedicine. This approach allows patients with suspected pneumonia to receive timely evaluation at home while providing a mechanism for referral to the ED when clinically necessary. Because downstream ED visits, hospitalization, readmission, and mortality were not assessed, the effectiveness or safety of this care model cannot be determined from the present study.

Patients referred to the ED had significantly higher CRB-65 scores than those managed without ED referral, demonstrating an association between documented illness severity and ED referral decisions. Although CRB-65 provides a standardized approach to pneumonia risk stratification, referral decisions in the present study also incorporated clinician judgment and patient-specific factors, including comorbidities. Prior studies have similarly emphasized that pneumonia severity scores should complement rather than replace clinical judgment when determining the appropriate site of care [24]. Future prospective studies should evaluate hybrid care models using standardized severity assessments together with longitudinal patient outcomes and patient-centered measures.

This study has several limitations. First, the small sample size, retrospective design, and single-region setting may limit the generalizability of the findings. Second, because this was a retrospective descriptive case-series study, we were unable to determine downstream clinical outcomes, including ED presentation, hospitalization, readmission, or mortality, precluding assessment of the safety or effectiveness of outpatient management. Additionally, ED referral decisions reflected clinician judgment and patient-specific factors rather than a standardized CRB-65–based referral protocol. Third, illness severity was assessed using the CRB-65 score because contemporaneous blood urea nitrogen measurements were not consistently available in the EHR. Although CRB-65 is an established tool for community-based pneumonia risk assessment, it may underestimate illness severity compared with CURB-65 in some patients. Despite these limitations, this study represents, to our knowledge, the first evaluation of a hybrid telemedicine–house-call model for pneumonia management. These findings provide preliminary evidence supporting this care model and may inform future multicenter prospective studies evaluating its effectiveness, scalability, and economic impact.

### Conclusions

This retrospective pilot study demonstrates the use of a hybrid telemedicine–house-call model for the outpatient evaluation of adults with pneumonia in a real-world clinical setting. Patients referred to the ED had higher CRB-65 scores than those managed without ED referral, demonstrating an association between higher documented CRB-65 scores and ED referral. Because this descriptive case-series study lacked a comparison group and did not assess downstream clinical outcomes, no conclusions can be drawn regarding the effectiveness, safety, or impact of the model on ED use. Prospective comparative studies with longitudinal follow-up are needed to evaluate these outcomes.

## Data Availability

The datasets generated and/or analyzed during this study are not publicly available due to institutional restrictions but are available from the corresponding author on reasonable request.

## Abbreviations

CRB-65: confusion, respiratory rate, blood pressure, age ≥65 years
CURB-65: confusion, urea, respiratory rate, blood pressure, age ≥65 years
ED: emergency department
EHR: electronic health record
ICD-10: International Classification of Diseases, 10th Revision
IRB: Institutional Review Board
